# Association Between cMIND Diet and Dementia Among Chinese Older Adults: A Population-Based Cross-Sectional Study

**DOI:** 10.3390/nu17223529

**Published:** 2025-11-11

**Authors:** Yu Zhang, Yuanyuan Lan, Youtao Mou, Yingjiao Deng, Ziyi Chen, Yandi Fu, Zumin Shi, Lei Zhang, Yong Zhao

**Affiliations:** 1 School of Public Health, Chongqing Medical University, Chongqing 400016, China; 3355622191@stu.cqmu.edu.cn (Y.Z.); 2024121736@stu.cqmu.edu.cn (Y.L.); 2024121761@stu.cqmu.edu.cn (Y.M.); 2023111615@stu.cqmu.edu.cn (Y.D.); 2 Research Center for Medicine and Social Development, Chongqing Medical University, Chongqing 400016, China; 3 Research Center for Public Health Security, Chongqing Medical University, Chongqing 400016, China; 4 Nutrition Innovation Platform-Sichuan and Chongqing, School of Public Health, Chongqing Medical University, Chongqing 400016, China; 5 College of Traditional Chinese Medicine, Chongqing Medical University, Chongqing 400016, China; 2022222391@stu.cqmu.edu.cn; 6 Faculty of Paediatrics, Chongqing Medical University, Chongqing 400016, China; 2022220323@stu.cqmu.edu.cn; 7 Department of Nutrition Sciences, College of Health Sciences, Qatar University, Doha 2713, Qatar; zumin@qu.edu.qa; 8 China-Australia Joint Research Center for Infectious Diseases, School of Public Health, Xi’an Jiaotong University Health Science Center, Xi’an 710061, China; lei.zhang1@monash.edu; 9 Artificial Intelligence and Modelling in Epidemiology Program, Melbourne Sexual Health Centre, Alfred Health, Melbourne 3053, Australia; 10 School of Translational Medicine, Faculty of Medicine, Nursing and Health Sciences, Monash University, Melbourne 3800, Australia; 11 Chongqing Key Laboratory of Child Nutrition and Health, Children’s Hospital of Chongqing Medical University, Chongqing 400014, China

**Keywords:** cMIND, aging, CLHLS, dementia, dietary pattern

## Abstract

Background: China’s rapidly aging population has led to a growing burden of dementia, marked by cognitive decline and heavy social and economic costs. Dietary patterns have been identified as a critical means for prevention. Methods: This study drew on data from the China Longitudinal Health and Longevity Survey (CLHLS). Three logistic regression models were applied to examine the association between the Chinese version of the Mediterranean-Dietary Approaches to Stop Hypertension Intervention for Neurodegenerative Delay (cMIND) diet and dementia. To test the stability of the results, we conducted two sensitivity analyses. Restricted cubic spline (RCS) models were used to assess the potential for a nonlinear relationship. Subgroup and interaction analyses were conducted to explore heterogeneity across covariates and main effects. Propensity score matching (PSM) was performed as a secondary analysis to minimize the influence of confounding factors. Results: The study included 9142 participants, with a dementia prevalence of 10.7% among Chinese older adults. After adjusting for all covariates, each one-unit increase in the cMIND diet score was associated with an 11% lower prevalence of dementia (OR = 0.89; 95% CI: 0.84–0.93). After full adjustment, the RCS model confirmed a significant and linear dose–response association between adherence to the cMIND diet and dementia. Comparable associations were observed across most subgroups. Conclusions: Adherence to the cMIND diet was significantly associated with a lower prevalence of dementia in Chinese older adults, with evidence of a clear dose–response effect. These findings highlight the potential of the cMIND diet as a preventive strategy against dementia in this population.

## 1. Introduction

As the global population ages, dementia has become an urgent public health issue worldwide. Dementia is defined as a syndrome involving significant cognitive decline that disrupts independent daily activities [1]. Globally, dementia is the seventh leading cause of death and a major source of disability and dependency in older adults [2]. In 2019, approximately 57.4 million individuals were living with dementia, and this number is projected to increase to 152.8 million by 2050 due to population growth and population aging [3]. In 2021, the prevalence of dementia in China was 1194.2 per 100,000 people, with a mortality rate of 34.6 per 100,000. The prevalence among older Chinese adults is expected to further rise [4]. Beyond cognitive deterioration and poorer quality of life, dementia imposes substantial social and economic burdens on families and communities. Although overall global mortality rates have declined, dementia still increases the risk of death by approximately threefold, and this relative risk has remained unchanged across two cohorts more than two decades apart [5]. The burden of dementia in China also shows substantial regional variation, closely linked to economic development, geographic location, and exposure to risk factors [6]. Among modifiable risk factors, dietary patterns have been identified as a crucial determinant in dementia prevention [7,8].

In recent years, there has been extensive research on the impact of traditional regional diets (i.e., dietary patterns from various regions around the world) on the prevention of non-communicable diseases [9]. Traditional regional diets not only benefit physical health but also represent a sustainable lifestyle, aligning with cultural and environmental diversity. The association between diet and cognitive health has become a major area of research focus [10]. The “microbiome–gut–brain axis” offers a key theoretical framework for understanding diet–brain interactions [11]. This bidirectional system connects gut microbiota with the central nervous system through neural, endocrine, immune, and metabolic pathways [12]. Evidence shows that dietary patterns can modulate gut microbiota and its metabolites, thereby influencing brain health and cognitive function, partly via the vagus nerve [13,14,15]. Diets rich in dietary fiber, polyphenols, and certain fatty acids have been shown to promote cognitive health in older adults [16]. The Mediterranean diet, which emphasizes fruits, vegetables, and olive oil, complemented by moderate alcohol intake [17], has attracted considerable attention for its potential to slow neurodegenerative disease progression, largely due to its abundance of polyphenols, unsaturated fatty acids, and antioxidants [18,19]. The Dietary Approaches to Stop Hypertension (DASH) diet, originally developed to manage hypertension, emphasizes low sodium and high potassium, calcium, and magnesium intake. The DASH diet encourages the consumption of fruits, vegetables, low-fat dairy, and whole grains while restricting that of saturated fats and cholesterol [20]. A systematic review and meta-analysis showed that strict adherence to the Mediterranean diet lowered the risk of progression from mild cognitive impairment to dementia by 33% [21]. Moreover, long-term adherence to the DASH diet has also been linked to better preservation of cognitive function in older adults [22]. Morris et al. [23] developed the Mediterranean-DASH Intervention for Neurodegenerative Delay (MIND) diet, integrating the Mediterranean and DASH patterns to highlight foods with neuroprotective potential. The MIND diet emphasizes natural, plant-based foods while limiting animal-derived products and those high in saturated fat. A cohort study in Australia reported that the cognitive benefits of the MIND diet were broadly generalizable [24].

However, the MIND dietary pattern emphasizes Western foods such as olive oil, deep-sea fish, and wine, which differ markedly from the traditional dietary habits of older Chinese adults [25]. To address this limitation, Huang et al. developed the Chinese version of the MIND (cMIND) diet based on the original MIND framework, adapting it to the characteristics of Chinese dietary patterns, and validated its applicability among older adults in China [26]. Its core components include whole grains, soy products, green tea, fresh fruits and vegetables, and low-salt cooking methods [26]. Several previous studies have examined the association between the cMIND diet and non-communicable diseases among older adults in China. For example, Adherence to the cMIND diet has been associated with the prevalence of hypertension, anxiety, and depression [27,28]. In addition, Huang X, Lin W, and their colleagues reported that moderate and high adherence to the cMIND diet was associated with a lower prevalence of cognitive impairment among Chinese older adults [26,29]. These findings provide further support for the potential benefits of the cMIND diet on cognitive function. However, given that dementia represents a more advanced stage along the continuum of cognitive decline, evidence regarding the association between the cMIND diet and dementia among Chinese older adults remains limited.

In conclusion, further studies are warranted to elucidate the association between the cMIND diet and dementia among older adults in China [30]. Therefore, this study explores the association between the cMIND diet and dementia in Chinese older adults, aiming to inform strategies for prevention and delay in this population.

## 2. Materials and Methods

### 2.1. Study Design and Population

The Chinese Longitudinal Healthy Longevity Survey (CLHLS) is a nationwide prospective cohort study of older adults in China. The survey aims to fill critical data and knowledge gaps in scientific research and policy analysis related to healthy aging in China [31]. From 1998 to 2018, eight survey waves were carried out in 23 provinces, municipalities, and autonomous regions. The study subjects of CLHLS were obtained through multi-stage stratified cluster sampling in 631 randomly selected cities and counties [32]. First, one eligible centenarian respondent was recruited in each selected city or county. Subsequently, one non-older adult, one octogenarian, and three older adults aged 65–79 years living in the same or nearby street, village, or town were matched to the centenarian. The age and sex of individuals aged 65–99 years were randomly assigned to ensure comparability with the randomly coded centenarians [33]. The survey collected comprehensive information on participants’ demographics, mental health, cognitive function, social participation, dietary behaviors, lifestyle habits, socioeconomic status, and family structure. All interviews and physical assessments were conducted one-on-one by trained interviewers to ensure data accuracy and reliability. The study was conducted in accordance with the Declaration of Helsinki, and approved by the Ethics Committee of Peking University (protocol code: IRB00001052-13074 and date of approval 8 August 2022), and all participants provided informed consent before data collection. Further details on the CLHLS can be accessed at https://opendata.pku.edu.cn/dataverse/CHADS (accessed on 8 August 2022).

The determination of the study’s sample size was based on the formula used for calculating sample sizes in cross-sectional research studies: [*n* = (Z^2^_α/2_pq)/δ^2^] [34], (1) n represents the sample size required for the study; (2) p denotes the prevalence rate of dementia in Chinese older adults; (3) q = (1 − p); (4) Z_α/2_ was set at 1.96 and α was set at 0.05 for a two-sided test; and (5) δ denotes the permissible error, calculated at 0.1 p. According to a previous study, the prevalence of dementia among older adults in China ranged from 2.6% to 13.5% [35]. Using the midpoint value of 8.1% for sample size estimation, the required minimum sample size for this study was calculated to be 4359 participants.

**Figure 1:**
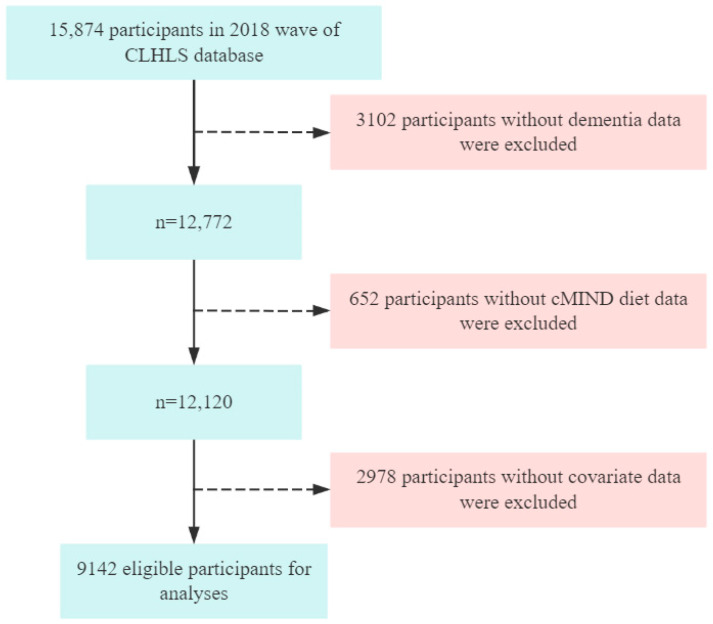
Flow chart of participant selection.

This analysis utilized cross-sectional data from the 2018 wave. Following the definition proposed by Livingston et al., older adults were defined as individuals aged 65 years and above [36]. The inclusion criteria for this study were as follows: (1) age ≥ 65 years; (2) completion of the 2018 assessments for the cMIND diet, dementia, and covariates; (3) voluntary participation in the study; and (4) ability to communicate normally with no family history of mental disorders. In this study, missing data were handled using case-wise deletion. The data cleaning process is illustrated in Figure 1. A total of 9142 eligible participants were included in the final analysis.

### 2.2. Measurement of Independent Variables

To reflect Chinese dietary habits, Huang et al. adapted the MIND diet into a culturally appropriate version for older Chinese adults [26]. The applicability of the cMIND diet in this population has been confirmed in multiple studies [29,37]. Dietary intake frequency was assessed using a food frequency questionnaire (FFQ). The cMIND diet includes 12 components: Types of staple food, amount of staple food, fresh vegetables, fresh fruit, cooking oil, fish, food made from beans, nut, mushroom or algae, garlic, tea and white sugar or candy. Types of staple food, amount of staple food and cooking oil were scored 0 or 1, while the other components were scored 0, 0.5, or 1. The detailed criteria are shown in Supplementary Table S1. The total cMIND diet score ranges from 0 to 12, with higher scores indicating greater adherence to the cMIND diet. We categorized the total scores of the cMIND diet into tertiles as follows: Lower (0–4), Medium (4.5–5.5), High (6–12).

### 2.3. Measurement of Dependent Variables

Dementia was defined based on the criteria of the Diagnostic and Statistical Manual of Mental Disorders (DSM)-5 and International Classification of Diseases 10th edition (ICD-10). Specifically, participants were classified as having dementia if they exhibited both cognitive impairment and functional impairment consistent with these diagnostic standards, or if they reported a physician’s diagnosis of dementia [38]. This definition has been validated by Liu et al. [39], demonstrating reliability in the Chinese older population.

Functional impairment was assessed using the Activities of Daily Living (ADL) scale, which includes six domains: bathing, dressing, toileting, indoor activities, continence, and eating. The total ADL score ranges from 6 to 18, with a score ≥ 7 indicating functional impairment [40].

Cognitive impairment was assessed with the Chinese version of the Mini-Mental State Examination (MMSE), covering orientation, registration, attention and calculation, recall, language, comprehension, and self-regulation abilities. The MMSE consists of 24 items, with a total score ranging from 0 to 30. A score of <18 indicating cognitive impairment [41].

Self-reported doctor-diagnosed dementia was determined through a two-step question process. Participants were first asked, “Do you currently have dementia?” If the answer was “yes” the interviewer followed up with, “Was this diagnosed by a hospital or physician?” If participants also answered “yes”, they were defined as having self-reported doctor-diagnosed dementia [42].

### 2.4. Measurement of Covariates

To control for potential confounding factors, we incorporated covariates encompassing participants’ demographic characteristics, lifestyle behaviors, and health conditions in the analyses. The demographic characteristics included age, gender, residence, marital status, economic status, education level and living arrangements. The lifestyle behaviors included smoking, drinking and exercise. The health conditions included hypertension, diabetes, heart disease, dyslipidemia and body mass index (BMI). More detailed information about these covariates can be found in Supplementary Table S2.

### 2.5. Statistical Analysis

The Kolmogorov–Smirnov test was applied to examine normality of continuous variables. Categorical variables are presented as numbers and percentages (n (%)). Continues variables are presented as median and interquartile range (M (Q_1_, Q_3_)). The chi-square test was used to evaluate group differences in categorical variables. Logistic regression models were employed to examine the association between the cMIND diet and dementia. A variance inflation factor of less than 5 was considered to indicate that there is no multicollinearity problem between the variables [43]. Three regression models were built: Model 1 was adjusted for age and gender; Model 2 was further adjusted for residence, marital status, education level, living arrangement, economic status, smoking, drinking and exercise based on Model 1; Model 3 further adjusted for hypertension, diabetes, heart disease, dyslipidemia and BMI based on Model 2. Two sensitivity analyses were carried out to test the robustness of the results: (1) missing covariate data were imputed using multiple chained equations, and (2) exclusion of participants with diabetes, hypertension, dyslipidemia, or heart disease. Using Model 3 with the continuous cMIND score, a restricted cubic spline (RCS) analysis was conducted to explore the potential non-linear association between the cMIND diet and dementia. Further subgroup analyses and interaction tests were conducted to examine the heterogeneity between covariates and primary variables, assessing potential effect modifications by these factors. To mitigate the potential for false positive errors arising from multiple comparisons, we adjusted the *p* values using the false discovery rate (FDR) method [44]. 

Propensity score matching (PSM) was applied to minimize confounding by balancing covariates across groups with similar propensity scores. A secondary analysis was then conducted based on the matched sample.

All analyses were conducted with SPSS 26.0 and R 4.3.0, and a two-tailed *p*-value < 0.05 was considered statistically significant.

## 3. Results

### 3.1. Basic Characteristics of Study Participants

The prevalence of dementia among Chinese older adults in the study was 10.7%. The final analytic sample comprised 9142 participants, including 5058 females (55.3%) and 4084 males (44.7%), with 5380 individuals (58.9%) aged 80 years or older. Table 1 shows significant differences between dementia and non-dementia in terms of age, gender, marital status, economic status, education level, smoking, drinking, exercise, living arrangements, BMI, hypertension, diabetes, and dyslipidemia (*p* < 0.05).

As presented in Table 2, participants were stratified into three groups based on cMIND diet tertiles (Lower, Medium and High), showing significant group differences in age, gender, residence, marital status, economic status, education level, smoking, drinking, exercise, living arrangements, BMI, hypertension, diabetes, heart disease and dyslipidemia (*p* < 0.05).

### 3.2. Association Between cMIND Diet and Dementia

Table 3 presents the results of the logistic regression models under different levels of covariate adjustment. After adjusting for gender, age, residence, marital status, education level, living arrangement, economic status, smoking, drinking, exercise, hypertension, diabetes, heart disease, dyslipidemia and BMI, each one-unit increase in the cMIND diet score was associated with an 11% lower prevalence of dementia (OR = 0.89; 95% CI: 0.84–0.93). Compared with participants in the lowest tertile of the cMIND diet score, those in the medium and high adherence groups had 18% (OR = 0.82; 95% CI: 0.69–0.97) and 24% (OR = 0.76; 95% CI: 0.61–0.94) lower odds of dementia, respectively.

**Figure 2:**
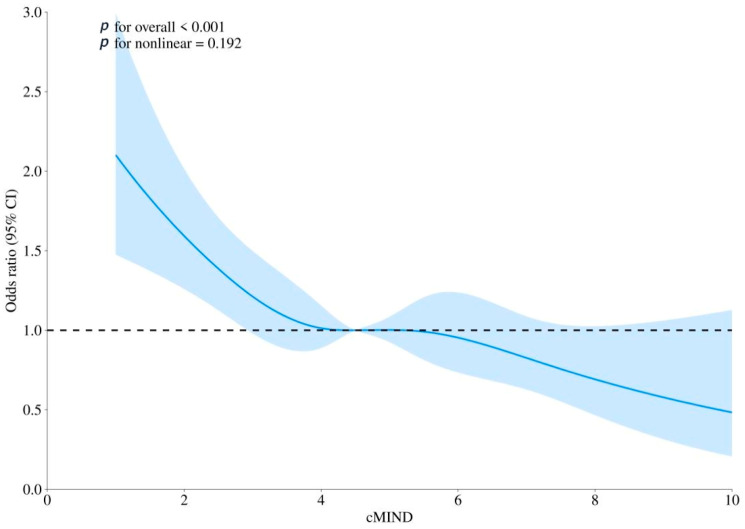
The association between cMIND diet with dementia using restricted cubic spline (RCS) regression analysis. Note: Model was adjusted for gender, age, residence, marital status, education level, living arrangement, economic status, smoking, drinking, exercise, hypertension, diabetes, heart disease, dyslipidemia, and body mass index. CI: confidence interval; OR: odds ratio. The solid blue line represents the estimated odds ratio of the outcome across varying levels of adherence to the cMIND diet, with the blue shaded area indicating the 95% confidence interval. The horizontal black dashed line represents the reference value (OR = 1.0).

The fully adjusted RCS model confirmed a significant association between cMIND diet and dementia (*p* for overall < 0.001), and a linear relationship was observed (non-linearity *p* = 0.192). Detailed results are presented in Figure 2.

### 3.3. Sensitivity Analysis

Two sensitivity analyses were conducted to confirm robustness: (1) Missing values of covariates were imputed using multiple chained equations to reduce bias caused by direct deletion. Supplementary Table S3 shows that each one-unite increase in the cMIND diet score was associated with an 8.0% reduction in the odds of dementia in Model 3 (OR = 0.92; 95% CI: 0.88–0.97). Compared to the low adherence group, high adherence to the cMIND diet was significantly protective against dementia in older adults. (2) Participants with diabetes, hypertension, dyslipidemia, or heart disease were excluded. Supplementary Table S4 indicates that, in Model 3, the cMIND diet consistently showed protective effects against dementia when modeled as either a continuous or categorical variable.

### 3.4. Subgroup and Interaction Analysis

**Figure 3:**
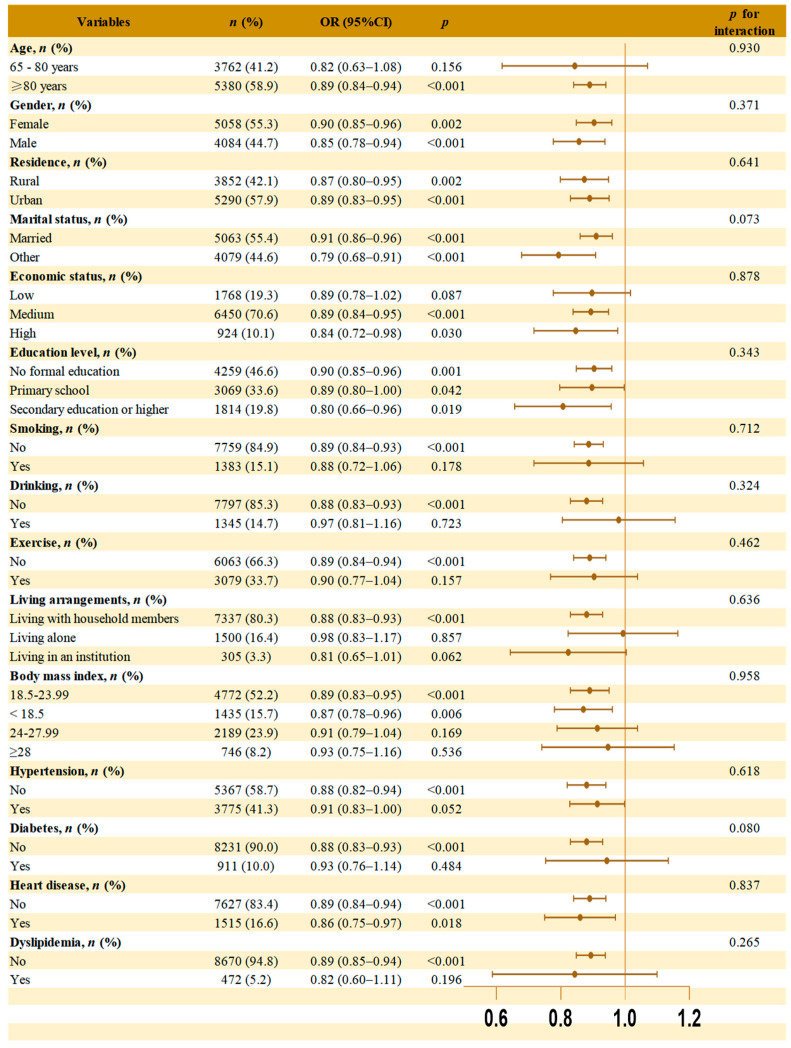
Subgroup analyses and interaction analysis of the association between cMIND diet and dementia. Note: Model was adjusted for gender, age, residence, marital status, education level, living arrangement, economic status, smoking, drinking, exercise, hypertension, diabetes, heart disease, dyslipidemia, and body mass index. The brown dot in each subgroup represent the OR values. The horizontal line through the dot represents the 95% confidence interval (95% CI). The orange vertical line indicates the reference value (OR = 1.0).

Subgroup analyses were performed across gender, age, residence, marital status, education level, living arrangement, economic status, smoking, drinking, exercise, hypertension, diabetes, heart disease, dyslipidemia and BMI to test robustness and population differences, with results shown in Figure 3.

The association between the cMIND diet and dementia remained statistically significant in several subgroups, including participants aged ≥80 years, with different gender, residence, marital status, education level, medium or high economic status, non-smoking, non-drinking, lack of exercise, living with household members, BMI ≤ 23.99, whether or not heart disease, and those without hypertension, diabetes, or dyslipidemia. No significant interaction was found between the subgroup variables and the association between the cMIND diet and dementia.

### 3.5. Propensity Score Matching

PSM (1:1 nearest neighbor matching algorithm and a caliper width of 0.05) was applied to match low and high adherence groups and reduce confounding effects. The probability density distributions before and after matching are shown in Supplementary Figure S1, and baseline characteristics of the matched sample are presented in Supplementary Table S5. All variables demonstrated standardized mean differences below 0.1, indicating acceptable balance between groups [45].

The association analysis after matching is summarized in Table 4. In the fully adjusted Model 3, high adherence was associated with 25.0% lower odds of dementia versus low adherence (OR = 0.75; 95% CI: 0.58–0.97).

## 4. Discussion

This study, using cross-sectional data from the 2018 CLHLS, investigated the association between the cMIND diet and dementia in 9142 Chinese older adults. After adjusting for multiple confounding factors, adherence to the cMIND diet was significantly associated with a lower risk of dementia. Specifically, compared with low adherence, moderate and high adherence were linked to 18.0% and 24.0% lower dementia risk, respectively. To test the stability of the results, we conducted two sensitivity analyses. RCS analysis demonstrated a significant linear relationship between cMIND diet and dementia. The association also remained significant across all subgroups, with no evidence of interaction. Finally, PSM was performed as a secondary analysis to minimize the influence of confounding factors.

### 4.1. The Prevalence and Pathogenesis of Diseases Among Older Adults in China

The prevalence of dementia among older adults in China is 10.7%, consistent with findings from Wang YQ [46], Liu Y [35], and others. Alzheimer’s disease (AD) is the most common form of dementia [47], and its pathogenesis has been the most extensively studied. Current research can be broadly summarized into three major hypotheses. First, the amyloid cascade hypothesis. Proposed by Hardy and Allsop in 1991 [47], this hypothesis suggests that the abnormal accumulation of β-amyloid (Aβ) plays a central role in AD development. Aβ, derived from the proteolytic cleavage of amyloid precursor protein (APP), progressively aggregates into neurotoxic plaques, leading to mitochondrial dysfunction, synaptic impairment, and neuronal death, thereby driving the onset and progression of AD [48]. Second, the hyperphosphorylated tau hypothesis. Tau, encoded by the MAPT gene [49], becomes pathologically altered through abnormal hyperphosphorylation, resulting in conformational changes and loss of microtubule-stabilizing function [50,51]. This process disrupts neuronal structure and function. Higher levels of tau phosphorylation are associated with greater aggregation and more severe AD pathology [52]. Third, the neuroinflammation hypothesis. Pathological stimuli and neuronal injury can induce pro-inflammatory responses [52]. Pro-inflammatory cytokines promote the release of additional inflammatory mediators and reactive oxygen species (ROS), while also upregulating complement system activity, thereby amplifying neurotoxic effects. The synergistic interaction between these cytokines and reactive astrocytes may activate destructive neuronal signaling pathways, promoting the deposition of Aβ plaques and the hyperphosphorylation of tau proteins, leading to further neuronal damage.

### 4.2. Mechanistic Pathways Association the cMIND Diet to Dementia

The cMIND diet, as a comprehensive dietary pattern, is characterized by its emphasis on a balanced intake of multiple nutrients and their synergistic interactions with lifestyle factors [26]. Our findings align with prior studies and further support the potential role of healthy eating in dementia prevention. Polyphenols, vitamin E, and n-3 polyunsaturated fatty acids, which are abundant in berries, leafy greens, and olive oil—key components of the MIND diet—can inhibit excessive microglial activation, reduce pro-inflammatory cytokine release, neutralize free radicals, and repair oxidative neuronal damage. These combined mechanisms contribute to reduced Aβ deposition and tau protein phosphorylation [53,54]. Moreover, the low-sodium principle of the DASH diet, together with the flavonoids present in the MIND diet, enhances vascular endothelial function, regulates blood pressure, lowers the risk of cerebrovascular lesions, and safeguards cerebral blood flow and Aβ clearance. Collectively, these effects contribute to a reduced risk of dementia [55].

### 4.3. The Association Between cMIND Diet and Dementia in Different Population Subgroups

This study systematically examined the associations between cMIND diet and dementia across different population subgroups, and consistent associations were observed. Specifically, in the subgroup aged ≥80 years, adherence to the cMIND diet was significantly associated with a lower prevalence of dementia. Among older adults, the brain typically accumulates pathological damage over decades. The cMIND diet’s rich anti-inflammatory components help promote a low-inflammation brain microenvironment, thereby slowing neurodegenerative processes and enhancing cognitive function [56]. Furthermore, this study found that rural residents benefited more from the cMIND diet than their urban counterparts. This suggests that preserving and promoting traditional dietary patterns may be crucial for maintaining neurological health in the context of rapid urbanization [57]. This study found that the protective effect of the cMIND diet against dementia was more pronounced among individuals with other marital statuses than among those who were married. Liu et al. found that individuals experiencing divorce, separation, widowhood, or remaining single exhibited a significantly higher risk of dementia compared with married individuals [22]. According to the marital resources model, marriage provides unique social, psychological, and economic resources that are difficult to obtain through other forms of relationships, thereby positively influencing health and longevity [58]. At the same time, socioeconomic disadvantage is a well-recognized risk factor for dementia [59]. Studies by French et al. and Hickey et al. have both found that individuals with lower income and education levels may face limitations in food choices and reduced health literacy [60,61]. Studies by Kivimäki et al. and Sanlier et al. have shown that individuals with lower socioeconomic status face a higher risk of dementia compared with those of higher status [62,63]. Furthermore, our findings indicate that living with household members was significantly associated with a lower prevalence of dementia, whereas no clear benefit was observed for those living alone. This observation underscores the synergistic role of social interaction in dietary interventions. Shared meals not only enhance adherence to healthy eating patterns but may also independently promote the development of cognitive reserve through social engagement. In the lifestyle dimension, this study found no significant protective effect of the cMIND diet against dementia among current smokers and drinkers. Tobacco and alcohol may diminish or even counteract the diet’s neuroprotective effects, consistent with the findings of Scarmeas et al. indicating that lifestyle and social support can modulate dietary outcomes [64]. Regarding health status, this study also found that adherence to the cMIND diet significantly lowered dementia risk among participants without hypertension or dyslipidemia. Compared with hypertensive individuals, those without hypertension exhibit healthier vascular function and maintain relatively stable cerebral blood flow. Additionally, the cMIND diet’s restriction of saturated and trans fats helps regulate lipid levels and slow the progression of atherosclerosis, thereby further improving cerebrovascular health [27]. No significant protective effect was observed among diabetic participants, whereas a risk reduction was evident in non-diabetic individuals. This finding aligns with the “metabolic–neurodegenerative axis” hypothesis, which suggests that chronic hyperglycemia and insulin resistance may undermine potential dietary benefits by promoting amyloid deposition, damaging the blood–brain barrier, and disrupting insulin signaling within the brain [65].

### 4.4. Advantages and Limitations

The results of this study provide new perspectives for public health intervention strategies, particularly among older adults in China. The cMIND diet, as a non-pharmacological preventive approach, holds promise as an important tool for alleviating the burden of dementia. These findings offer scientific evidence for public health policymakers and support the implementation of cognitive function-focused dietary interventions, carrying significant public health value and practical implications. This study also has several limitations. First, this analysis was based on cross-sectional data, which limits the ability to establish a causal association between the cMIND diet and dementia and does not rule out the possibility of reverse causation. The findings of this study can only support an association between the cMIND diet and dementia. Future prospective cohort studies are needed to clarify the causality and its directionality. Second, although we have controlled for potential confounders as thoroughly as possible, the influence of other unmeasured factors, such as environmental exposures or genetic predispositions, cannot be entirely ruled out. Third, both the cMIND diet and dementia were assessed via self-report. Although data collectors received rigorous training, the possibility of social desirability and recall biases cannot be ruled out. Future studies should adopt objective measures whenever possible to minimize the impact of such potential biases. Finally, the database used in this study is specific to the Chinese population. Given the differences in diet and culture across regions globally, the applicability and generalizability of the cMIND diet may be limited. Future research should prioritize cross-cultural comparative studies to examine the effects of different dietary patterns in diverse populations. Such an approach would facilitate the development of more targeted dietary recommendations for dementia prevention on a global scale.

## 5. Conclusions

The findings suggest that greater adherence to the cMIND diet is associated with a lower prevalence of dementia among Chinese older adults, with a clear dose–response relationship. Further subgroup analyses confirm that this association persists across different demographic subgroups. The findings of this study highlight the potential of the cMIND diet in preventing dementia among older Chinese adults, indicating the need for larger cohort studies or randomized controlled trials to strengthen causal inference, and offering innovative strategies to reduce the dementia burden through localized dietary interventions.

## Figures and Tables

**Table 1:** Baseline characteristics of study population by dementia.

Variables	Total (*n* = 9142)	No Dementia (*n* = 8167)	Dementia (*n* = 975)	Statistic	*p*-Value
Age, *n* (%)				χ^2^ = 653.3	<0.001
65–80 years	3762 (41.2)	3732 (99.2)	30 (0.8)		
≥80 years	5380 (58.9)	4435 (82.4)	945 (17.6)		
Gender, *n* (%)				χ^2^ = 106.7	<0.001
Female	5058 (55.3)	4367 (86.3)	691 (13.7)		
Male	4084 (44.7)	3800 (93.1)	284 (7.00)		
Residence, *n* (%)				χ^2^ = 0.2	0.690
Rural	3852 (42.1)	3447 (89.5)	405 (10.5)		
Urban	5290 (57.9)	4720 (89.2)	570 (10.8)		
Marital status, *n* (%)				χ^2^ = 484.8	<0.001
Married	5063 (55.4)	4200 (83.0)	863 (17.1)		
Other	4079 (44.6)	3967 (97.3)	112 (2.8)		
Economic status, *n* (%)				χ^2^ = 39.5	<0.001
Low	1768 (19.3)	1638 (92.7)	130 (7.4)		
Medium	6450 (70.6)	5744 (89.1)	706 (11.0)		
High	924 (10.1)	785 (85.0)	139 (15.0)		
Education level, *n* (%)				χ^2^ = 342.6	<0.001
No formal education	4259 (46.6)	3535 (83.0)	724 (17.0)		
Primary school	3069 (33.6)	2884 (94.0)	185 (6.0)		
Secondary education or higher	1814 (19.8)	1748 (96.4)	66 (3.6)		
Smoking, *n* (%)				χ^2^ = 56.5	<0.001
No	7759 (84.9)	6852 (88.3)	907 (11.7)		
Yes	1383 (15.1)	1315 (95.1)	68 (4.9)		
Drinking, *n* (%)				χ^2^ = 50.7	<0.001
No	7797 (85.3)	6891 (88.4)	906 (11.6)		
Yes	1345 (14.7)	1276 (94.9)	69 (5.1)		
Exercise, *n* (%)				χ^2^ = 270.4	<0.001
No	6063 (66.3)	5187 (85.6)	876 (14.5)		
Yes	3079 (33.7)	2980 (96.8)	99 (3.2)		
Living arrangements, *n* (%)				χ^2^ = 105.2	<0.001
Living with household members	7337 (80.3)	6520 (88.9)	817 (11.1)		
Living alone	1500 (16.4)	1417 (94.5)	83 (5.5)		
Living in an institution	305 (3.3)	230 (75.4)	75 (24.6)		
BMI, *n* (%)				χ^2^ = 187.6	<0.001
18.5–23.99	4772 (52.2)	4277 (89.6)	495 (10.4)		
<18.5	1435 (15.7)	1147 (79.9)	288 (20.1)		
24–27.99	2189 (23.9)	2055 (93.9)	134 (6.1)		
≥28	746 (8.2)	688 (92.2)	58 (7.8)		
Hypertension, *n* (%)				χ^2^ = 43.3	<0.001
No	5367 (58.7)	4699 (87.6)	668 (12.5)		
Yes	3775 (41.3)	3468 (91.9)	307 (8.1)		
Diabetes, *n* (%)				χ^2^ = 15.8	<0.001
No	8231 (90.0)	7318 (88.9)	913 (11.1)		
Yes	911 (10.0)	849 (93.2)	62 (6.8)		
Heart disease, *n* (%)				χ^2^ = 1.7	0.189
No	7627 (83.4)	6828 (89.5)	799 (10.5)		
Yes	1515 (16.6)	1339 (88.4)	176 (11.6)		
Dyslipidemia, *n* (%)				χ^2^ = 7.9	0.005
No	8670 (94.8)	7727 (89.1)	943 (10.9)		
Yes	472 (5.2)	440 (93.2)	32 (6.8)		

Notes: χ^2^: Chi-square test; BMI: body mass index.

**Table 2:** Baseline characteristics of study population by cMIND diet.

Variables	Lower (0–4) (*n* = 3560)	Medium (4.5–5.5) (*n* = 3111)	High (6–12) (*n* = 2471)	Statistic	*p*-Value
Age, *n* (%)				χ^2^ = 322.9	<0.001
65–80 years	1099 (29.2)	1333 (35.4)	1330 (35.4)		
≥80 years	2461 (45.7)	1778 (33.1)	1141 (21.2)		
Gender, *n* (%)				χ^2^ = 151.9	<0.001
Female	2215 (43.8)	1701 (33.6)	1142 (22.6)		
Male	1345 (32.9)	1410 (34.5)	1329 (32.5)		
Residence, *n* (%)				χ^2^ = 217.2	<0.001
Rural	1734 (45.0)	1376 (35.7)	742 (19.3)		
Urban	1826 (34.5)	1735 (32.8)	1729 (32.7)		
Marital status, *n* (%)				χ^2^ = 340.7	<0.001
Married	2335 (46.1)	1700 (33.6)	1028 (20.3)		
Other	1225 (30.0)	1411 (34.6)	1443 (35.4)		
Economic status, *n* (%)				χ^2^ = 402.6	<0.001
Low	442 (25.0)	714 (40.4)	612 (34.6)		
Medium	2567 (39.8)	1650 (25.6)	2233 (34.6)		
High	551 (59.6)	107 (11.6)	266 (28.8)		
Education level, *n* (%)				χ^2^ = 993.4	<0.001
No formal education	2167 (50.9)	671 (15.8)	1421 (33.4)		
Primary school	1072 (34.9)	867 (28.3)	1130 (36.8)		
Secondary education or higher	321 (17.7)	933 (51.4)	560 (30.9)		
Smoking, *n* (%)				χ^2^ = 6.5	0.039
No	3064 (39.5)	2616 (33.7)	2079 (26.8)		
Yes	496 (35.9)	495 (35.8)	392 (28.3)		
Drinking, *n* (%)				χ^2^ = 39.6	<0.001
No	3135 (40.2)	2624 (33.7)	2038 (26.1)		
Yes	425 (31.6)	487 (36.2)	433 (32.2)		
Exercise, *n* (%)				χ^2^ = 473.6	<0.001
No	2738 (45.2)	2089 (34.5)	1236 (20.4)		
Yes	822 (26.7)	1022 (33.2)	1235 (40.1)		
Living arrangements, *n* (%)				χ^2^ = 99.0	<0.001
Living with household members	2730 (37.2)	2076 (28.3)	2531 (34.5)		
Living alone	734 (48.9)	286 (19.1)	480 (32.0)		
Living in an institution	96 (31.5)	109 (35.7)	100 (32.8)		
BMI, *n* (%)				χ^2^ = 243.7	<0.001
18.5–23.99	1934 (40.5)	1653 (34.6)	1185 (24.8)		
<18.5	727 (50.7)	460 (32.1)	248 (17.3)		
24–27.99	659 (30.1)	756 (34.5)	774 (35.4)		
≥28	240 (32.2)	242 (32.4)	264 (35.4)		
Hypertension, *n* (%)				χ^2^ = 113.1	<0.001
No	2297 (42.8)	1814 (33.8)	1256 (23.4)		
Yes	1263 (33.5)	1297 (34.4)	1215 (32.2)		
Diabetes, *n* (%)				χ^2^ = 152.4	<0.001
No	3335 (40.5)	2819 (34.3)	2077 (25.2)		
Yes	225 (24.7)	292 (32.1)	394 (43.3)		
Heart disease, *n* (%)				χ^2^ = 57.9	<0.001
No	3046 (39.9)	2639 (34.6)	1942 (25.5)		
Yes	514 (33.9)	472 (31.2)	529 (34.9)		
Dyslipidemia, *n* (%)				χ^2^ = 73.2	<0.001
No	3435 (39.6)	2970 (34.3)	2265 (26.1)		
Yes	125 (26.5)	141 (29.9)	206 (43.6)		

Notes: χ^2^: Chi-square test; BMI: body mass index; cMIND: the Chinese version of the Mediterranean-DASH intervention for neurodegenerative delay.

**Table 3:** Association between cMIND diet with dementia. (*n* = 9142).

Variables	Model 1		Model 2		Model 3	
	OR (95% CI)	*p*	OR (95% CI)	*p*	OR (95% CI)	*p*
cMIND diet was used as a continuous variable	0.85 (0.81, 0.89)	<0.001	0.88 (0.84, 0.93)	<0.001	0.89 (0.84, 0.93)	<0.001
cMIND diet was used as a categorical variable (vs. Lower (0–4))						
Medium (4.5–5.5)	0.74 (0.63, 0.87)	<0.001	0.80 (0.68, 0.94)	0.008	0.82 (0.69, 0.97)	0.017
High (6–12)	0.60 (0.49, 0.73)	<0.001	0.74 (0.60, 0.92)	0.006	0.76 (0.61, 0.94)	0.012

Notes: cMIND: the Chinese version of the Mediterranean-DASH intervention for neurodegenerative delay. Model 1 controlling for gender and age. Model 2 controlling for gender and age, residence, marital status, education level, living arrangement, economic status, smoking, drinking, and exercise. Model 3 controlling for gender and age, residence, marital status, education level, living arrangement, economic status, smoking, drinking, exercise, hypertension, diabetes, heart disease, dyslipidemia, and body mass index.

**Table 4:** Association analysis between cMIND diet and dementia after propensity score matching (*n* = 3250).

Variable	Model 1		Model 2		Model 3	
	OR (95% CI)	*p*	OR (95% CI)	*p*	OR (95% CI)	*p*
cMIND diet was used as a categorical variable (vs. Lower)						
High	0.85 (0.81, 0.89)	<0.001	0.76 (0.59, 0.98)	0.032	0.75 (0.58, 0.97)	0.026

Notes: cMIND: the Chinese version of the Mediterranean-DASH intervention for neurodegenerative delay. Model 1 controlling for gender and age. Model 2 controlling for gender and age, residence, marital status, education level, living arrangement, economic status, smoking, drinking, and exercise. Model 3 controlling for gender and age, residence, marital status, education level, living arrangement, economic status, smoking, drinking, exercise, hypertension, diabetes, heart disease, dyslipidemia, and body mass index.

## Data Availability

The original data presented in the study are openly available in http://opendata.pku.edu.cn/ (accessed on 8 August 2022).

## Supplementary Materials

The following supporting information can be downloaded at: https://www.mdpi.com/article/10.3390/nu17223529/s1, Table S1: The value assignment of cMIND diet, Table S2: The value assignment of covariates, Table S3: Association between cMIND diet and dementia: A Multiple Imputation by Chained Equations Approach, Table S4: Association between cMIND diet and dementia, except hypertension, diabetes, heart disease and dyslipidemia, Table S5: Baseline characteristics before and after propensity score matching, Figure S1: Covariates matching effect.
