# Supplementary material for: Association Between cMIND Diet and Dementia Among Chinese Older Adults: A Population-Based Cross-Sectional Study
Source: Nutrients. 2025 Nov 11;17(22):3529. doi: 10.3390/nu17223529 (PMC12655610; doi:10.3390/nu17223529)
Supplement: Supplementary file 1 [file nutrients-17-03529-s001.zip › nutrients-3963554-supplementary.pdf]

Supplementary Materials

Table S1. The value assignment of cMIND diet

Components	Score		
	**0**	**0.5**	**1**
Types of staple food	Rice / Whear	/	Whole grains
Amount of staple food	<250g or >400g	/	250g–400g
Fresh vegetables	≤2 servings/week	3−5/week	≥6 servings/week
Fresh fruit	≤2 servings/week	3−5/week	≥6 servings/week
Cooking oil	Animal oil	/	vegetable oil
Fish	<1/month	1−3/month	≥1 meal/week
Food made from beans	<1 meal/week	1−3/week	≥4 meals/week
Nut	<1 serving/week	1−4/week	≥5 servings/week
Mushroom or algae	≤1 meal/week	1−3/week	≥4 meals/week
Garlic	<1 meal/week	1−3/week	≥4 meals/week
Tea	Not almost every day	Other types of tea (almost every day)	Green tea (almost every day)
White sugar or candy	≥2 servings/week	1/month−1/week	<1 serving/month

Table S2. The value assignment of covariates

Variable	Assignment
Age	0 = 65–80 years, 1 = ≥ 80 years
Gender	0 = Female, 1 = Male
Residence	0 = Rural, 1 = Urban
Marital status	0 = Married, 1 = Other
Economic status	0 = Low, 1 = Medium, 2 = High
Education level	0 = No formal education, 1 = Primary school, 2 = Secondary education or higher
Smoking	0 = No, 1 = Yes
Drinking	0 = No, 1 = Yes
Exercise	0 = No, 1 = Yes
Living arrangements	0 = Living with household members, 1 = Living alone, 2 = Living in an institution
Hypertension	0 = No, 1 = Yes
Diabetes	0 = No, 1 = Yes
Heart disease	0 = No, 1 = Yes
Dyslipidemia	0 = No, 1 = Yes
BMI	0 = 18.5–23.99, 1 = < 18.5, 2 = 24–27.99, 3 = ≥28

Table S3. Association between cMIND diet and dementia: A Multiple Imputation by Chained Equations Approach

Variable	Model 1		Model 2		Model 3	
	OR (95%CI)	*p*	OR (95%CI)	*p*	OR (95%CI)	*p*
cMIND diet was used as a continous variable	0.91(0.87,0.95)	<0.001	0.92(0.88,0.97)	0.006	0.92(0.88,0.97)	0.009
cMIND diet was used as a categorical variable						
Lower	Ref.		Ref.		Ref.	
Medium	0.85(0.73,0.99)	0.031	0.86(0.73,1.00)	0.099	0.86(0.73,1.01)	0.091
High	0.79(0.65,0.95)	0.022	0.81(0.66,0.99)	0.029	0.80(0.65,0.98)	0.024

Notes: cMIND: the Chinese version of the Mediterranean-DASH intervention for neurodegenerative delay.

Model 1 controlling for gender and age.

Model 2 controlling for gender and age, residence, marital status, education level, living arrangement, economic status, smoking, drinking, and exercise.

Model 3 controlling for gender and age, residence, marital status, education level, living arrangement, economic status, smoking, drinking, exercise, hypertension, diabetes, heart disease, dyslipidemia, and body mass index.

Table S4. Association between cMIND diet and dementia, except hypertension, diabetes, heart disease and dyslipidemia

Variable	Model 1		Model 2		Model 3	
	OR (95%CI)	*p*	OR (95%CI)	*p*	OR (95%CI)	*p*
cMIND diet was used as a continous variable	0.89(0.83,0.95)	0.006	0.91(0.84,0.97)	0.005	0.91(0.84,0.97)	0.006
cMIND diet was used as a categorical variable						
Lower	Ref.		Ref.		Ref.	
Medium	0.72(0.57,0.90)	0.004	0.76(0.60,0.96)	0.020	0.76(0.61,0.96)	0.014
High	0.74(0.55,0.98)	0.021	0.82(0.61,1.12)	0.089	0.82(0.61,1.12)	0.012

Notes: MIND: the Chinese version of the Mediterranean-DASH intervention for neurodegenerative delay.

Model 1 controlling for gender and age.

Model 2 controlling for gender and age, residence, marital status, education level, living arrangement, economic status, smoking, drinking, and exercise.

Model 3 controlling for gender and age, residence, marital status, education level, living arrangement, economic status, smoking, drinking, exercise and body mass index.

Table S5. Baseline characteristics before and after propensity score matching

Variable	Before PSM						After PSM					
	Total (n = 6031)	Lower (n = 3560)	High (n = 2471)	Statistic	*P*	SMD	Total (n = 3250)	Lower (n = 1625)	High (n = 1625)	Statistic	*p*	SMD
Age, n (%)				χ^2=319.5	<0.001					χ^2=0.2	0.697	
65−80 years	2429 (40.3)	1099 (30.9)	1330 (53.8)			0.460	1427 (43.9)	708 (43.6)	719 (44.3)			0.014
⩾80 years	3602 (59.7)	2461 (69.1)	1141 (46.2)			0.460	1823 (56.1)	917 (56.4)	906 (55.8)			0.014
Gender, n (%)				χ^2=151.4	<0.001					χ^2=0.1	0.725	
Female	3357 (55.7)	2215 (62.2)	1142 (46.2)			0.321	1712 (52.7)	861 (53.0)	851 (52.4)			0.012
Male	2674 (44.3)	1345 (37.8)	1329 (53.8)			0.321	1538 (47.3)	764 (47.0)	774 (47.6)			0.012
Residence, n (%)				χ^2=210.3	<0.001					χ^2=0.6	0.430	
Rural	2476 (41.1)	1734 (48.7)	742 (30.0)			0.408	1292 (39.8)	657 (40.4)	635 (39.1)			0.028
Urban	3555 (59.0)	1826 (51.3)	1729 (70.0)			0.408	1958 (60.3)	968 (59.6)	990 (60.9)			0.028
Marital status, n (%)				χ^2=340.2	<0.001					χ^2=0.2	0.648	
Married	3363 (55.8)	2335 (65.6)	1028 (41.6)			0.487	1717 (52.8)	865 (53.2)	852 (52.4)			0.016
Other	2668 (44.2)	1225 (34.4)	1443 (58.4)			0.487	1533 (47.2)	760 (46.8)	773 (47.6)			0.016
Economic status, n (%)				χ^2=378.7	<0.001					χ^2=0.1	0.949	
Low	1156 (19.2)	442 (12.4)	714 (28.9)			0.364	665 (20.5)	336 (20.7)	329 (20.3)			0.011
Medium	4217 (69.9)	2567 (72.1)	1650 (66.8)			0.113	2378 (73.2)	1185 (72.9)	1193 (73.4)			0.011
High	658 (10.9)	551 (15.5)	107 (4.3)			0.548	207 (6.4)	104 (6.4)	103 (6.3)			0.003

Education level, n (%)				χ^2=943.1	<0.001					χ^2=0.1	0.972	
No formal education	2838 (47.1)	2167 (60.9)	671 (27.2)			0.758	1304 (40.1)	655 (40.3)	649 (39.9)			0.008
Primary school	1939 (32.2)	1072 (30.1)	867 (35.1)			0.104	1346 (41.4)	672 (41.4)	674 (41.5)			0.002
Secondary education or higher	1254 (20.8)	321 (9.0)	933 (37.8)			0.593	600 (18.5)	298 (18.3)	302 (18.6)			0.006
Smoking, n (%)				χ^2=4.3	0.037					χ^2=0.6	0.450	
No	5143 (85.3)	3064 (86.1)	2079 (84.1)			0.053	2712 (83.5)	1364 (83.9)	1348 (83.0)			0.026
Yes	888 (14.7)	496 (13.9)	392 (15.9)			0.053	538 (16.6)	261 (16.1)	277 (17.1)			0.026
Drinking, n (%)				χ^2=37.3	<0.001					χ^2=2.4	0.119	
No	5173 (85.8)	3135 (88.1)	2038 (82.5)			0.147	2754 (84.7)	1393 (85.7)	1361 (83.8)			0.053
Yes	858 (14.2)	425 (11.9)	433 (17.5)			0.147	496 (15.3)	232 (14.3)	264 (16.3)			0.053
Exercise, n (%)				χ^2=469.3	<0.001					χ^2=0.0	0.913	
No	3974 (65.9)	2738 (76.9)	1236 (50.0)			0.538	2069 (63.7)	1033 (63.6)	1036 (63.8)			0.004
Yes	2057 (34.1)	822 (23.1)	1235 (50.0)			0.538	1181 (36.3)	592 (36.4)	589 (36.3)			0.004
Living arrangements, n (%)				χ^2=93.0	<0.001					χ^2=0.9	0.633	
Living with household members	4806 (79.7)	2730 (76.7)	2076 (84.0)			0.200	2672 (82.2)	1346 (82.8)	1326 (81.6)			0.032
Living alone	1020 (16.9)	734 (20.6)	286 (11.6)			0.283	453 (13.9)	220 (13.5)	233 (14.3)			0.023
Living in an institution	205 (3.4)	96 (2.7)	109 (4.4)			0.083	125 (3.9)	59 (3.6)	66 (4.1)			0.022
BMI, n (%)				χ^2=236.6	<0.001					χ^2=1.4	0.717	
18.5-23.99	3119 (51.7)	1934 (54.3)	1185 (48.0)			0.127	1706 (52.5)	857 (52.7)	849 (52.3)			0.010
< 18.5	975 (16.2)	727 (20.4)	248 (10.0)			0.346	449 (13.8)	228 (14.0)	221 (13.6)			0.013
24-27.99	1433 (23.8)	659 (18.5)	774 (31.3)			0.276	790 (24.3)	397 (24.4)	393 (24.2)			0.006

≥28	504 (8.4)	240 (6.7)	264 (10.7)			0.128	305 (9.4)	143 (8.8)	162 (10.0)			0.039
Hypertension, n (%)				χ^2=113.0	<0.001					χ^2=0.1	0.750	
No	3553 (58.9)	2297 (64.5)	1256 (50.8)			0.274	1845 (56.8)	927 (57.1)	918 (56.5)			0.011
Yes	2478 (41.1)	1263 (35.5)	1215 (49.2)			0.274	1405 (43.2)	698 (43.0)	707 (43.5)			0.011
Diabetes, n (%)				χ^2=146.7	<0.001					χ^2=0.6	0.457	
No	5412 (89.7)	3335 (93.7)	2077 (84.1)			0.263	2909 (89.5)	1461 (89.9)	1448 (89.1)			0.026
Yes	619 (10.3)	225 (6.3)	394 (15.9)			0.263	341 (10.5)	164 (10.1)	177 (10.9)			0.026
Heart disease, n (%)				χ^2=49.5	<0.001					χ^2=1.5	0.216	
No	4988 (82.7)	3046 (85.6)	1942 (78.6)			0.170	2669 (82.1)	1348 (83.0)	1321 (81.3)			0.043
Yes	1043 (17.3)	514 (14.4)	529 (21.4)			0.170	581 (17.9)	277 (17.1)	304 (18.7)			0.043
Dyslipidemia, n (%)				χ^2=65.5	<0.001					χ^2=0.7	0.405	
No	5700 (94.5)	3435 (96.5)	2265 (91.7)			0.175	3065 (94.3)	1,538 (94.7)	1527 (94.0)			0.028
Yes	331 (5.5)	125 (3.5)	206 (8.3)			0.175	185 (5.7)	87 (5.4)	98 (6.0)			0.028

Notes: χ^2: Chi-square test; BMI: body mass index

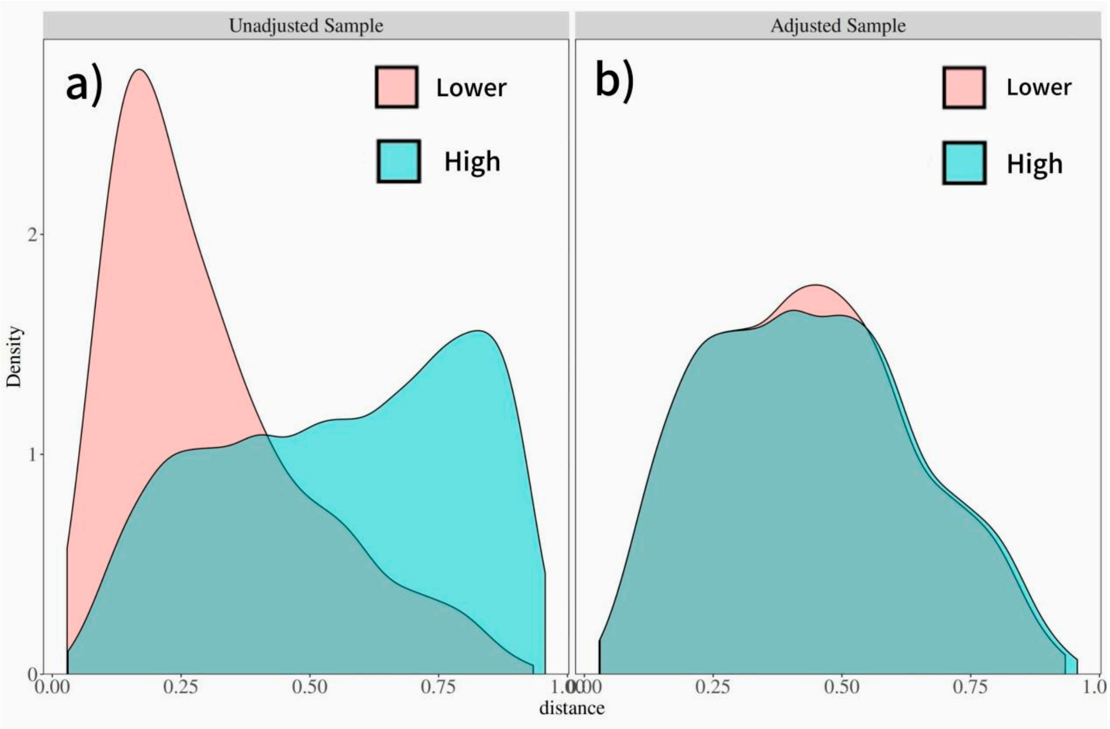

Figure S1. Covariates matching effect.

a) Before matching; b) After matching
